# Psychometric properties of a modified cultural awareness scale for use in higher education within the health and social care fields

**DOI:** 10.1186/s12909-020-02326-8

**Published:** 2020-11-06

**Authors:** Christine Kumlien, Melanie Bish, Engle A. Chan, Lynn Rew, P. S. Chan, Doris Leung, Elisabeth Carlson

**Affiliations:** 1 Department of Care Science, Malmö University, Jan Waldenströms gata 25, 20506 Malmö, Sweden; 2 Department of Cardiothoracic and Vascular Surgery, Skåne University Hospital, Malmö, Sweden; 3 Department of Rural Nursing & Midwifery, La Trobe Rural Health School, Melbourne, Australia; 4 School of Nursing, Hong Kong Polytechnic University, Hong Kong, Hong Kong; 5 School of Nursing, University of Texas at Austin, Austin, USA

**Keywords:** Cultural awareness, Cultural competence, Cultural awareness scale, Factor analysis

## Abstract

**Background:**

Cultural awareness and cultural competence have become important skills in higher education as populations continue to grow in diversity around the world. However, currently, there are few instruments designed to assess student awareness of the aspects of culture, and the existing instruments need further development and testing for use with different target populations. Therefore, the aim of this study was to test the psychometric properties of a modified version of the Cultural Awareness Scale (CAS) for use in higher education within the health and social care fields.

**Methods:**

A modified version of the CAS was developed, which was tested psychometrically using cross-sectional data. In total, 191 undergraduate students from different health and social care undergraduate programs in Sweden and Hong Kong responded to a call to test the modified instrument.

**Results:**

The results showed that the modified CAS is a four-factor measure of cultural awareness and possesses satisfactory internal consistency. Results also support the use of the modified CAS as a generic tool to measure cultural awareness among students in higher education within the health and social care fields.

**Conclusion:**

The modified CAS showed satisfactory psychometric properties and can be recommended as a generic tool to measure cultural awareness among students in higher education within the health and social care fields. However, further psychometric testing on the effectiveness of the modified CAS as a tool to evaluate the efficacy of cultural awareness interventions is required.

## Background

Cultural awareness and cultural competence have become important skills in higher education within the health and social care fields as populations grow in diversity around the world as a result of increased migration. For example, in the United States 2016, more than 40 million people are reported to be born in another country [1]. In Australia, in 2018, 29% of the estimated resident population were born overseas [2]. Further, in Sweden, in the same year (2018), almost 19% of the population was foreign-born [3]. As a result of this growing diversity, professionals in health care and social work are encountering more and more patients/clients from different cultures [4, 5]. Providing sufficient care and support requires in-depth knowledge and awareness among professionals working in inter-cultural settings. Consequently, higher education institutions have the responsibility to produce culturally aware graduates who are sensitive in their cross-cultural interactions [6]. However, instruments to assess student and faculty cultural awareness and the implications for practice are few. Moreover, existing instruments may need to be further developed and tested to fit different target populations.

### Conceptual framework

Culture can be defined as the values, beliefs, and norms that guide the thinking and actions of specific groups [7]. Further, culture helps individuals adapt to their environment [8]. Cultural competence is a systematic approach focusing on the ability of providers and organizations to effectively deliver health care services that meet the social, cultural, and linguistic needs of patients/clients [9] and can be understood as a process, not an event [4]. A more recent definition explains cultural competence as “using one’s understanding to represent and tailor health care that is equitable and ethical after becoming aware of oneself and others in a diverse cultural encounter” [10]. Conceptually, the literature suggests that cultural competence consists of several components such as cultural awareness, knowledge, skills, and sensitivity [4, 6, 10, 11]. Cultural awareness could be described as the recognition of one’s own cultural and professional background including beliefs, attitudes and behaviors [6]. Cultural knowledge is the process of seeking and obtaining an educational foundation about diverse cultural and ethnic groups which helps us understand the client’s worldview. Cultural skills refer to one’s ability to gather relevant cultural data regarding the client’s concerns and one’s accurate performance of a culturally-based assessment [4]. Cultural sensitivity means to have knowledge about differences and similarities between and among cultures without values assigned or judgements of those differences [11]. However, it has also been suggested that there is a hierarchy of cultural competence beginning with knowledge (the cognitive component), followed by awareness (the affective component) and sensitivity (the attitudinal component) [11]. This means to continually develop cultural competence, one needs to move from having knowledge followed by gaining awareness to being sensitive.

More recently, scholars have challenged the hierarchy of the cultural competence and its continuum. Curtis et al. [12] conducted an extensive literature review and concluded that healthcare practices would be more equitable with a transformative shift from “cultural competence” to “cultural safety.” Cultural safety focuses on examining power imbalances within the healthcare setting with the goal of approaching equity. Examining power imbalances within the healthcare setting includes critical appraisal of one’s own biases and stereotypes (p. 14). Cultural awareness, including this critique of an individual’s biases and stereotypes, is thereby a first step toward developing cultural safety and a critical component to address in higher education and research [13]. Consequently, valid and reliable instruments to assess the efficacy of cultural awareness enhancement interventions continue to be increasingly important.

### Literature review

There are several different instruments aimed at evaluating cultural competence in clinical and organizational settings. For example, 11 instruments developed to assess cultural competence in nurses and nursing students were found in a review by Loftin et al. [14]. The review revealed that the majority of the 11 instruments measured general cultural aspects and did not make distinctions between the different cultural groups [14]. Another review, by Lin et al. [15], found 10 instruments developed for measuring cultural competence in health care providers. Out of the 10 instruments found, only five were in English. The instruments evaluated different aspects of cultural competence. Two instruments used distinct perspectives to measure cultural awareness: The Cultural Awareness Scale (CAS) and the Nurse Cultural Competence Scale. Of these two instruments, CAS was considered to be the most appropriate for assessing cultural awareness [15]. Both Loftin et al. [14] and Lin et al. [15] concluded that current instruments lack an operational definition of cultural competence [15].

The concept of cultural competence is difficult to measure objectively [14], which makes it challenging for researchers to select the best instrument for their research purposes. Given that prior instruments for measuring cultural awareness were predominately tested in a single discipline, another important question is whether existing instruments are generic and can be used in different target groups to further expand our knowledge of their cultural awareness and cultural safety. If so, rigorous psychometric tests and retests of existing instruments are needed.

Rew et al. [6], the developers of the CAS, identified five categories of cultural awareness. The categories were based on a literature review of cultural awareness, cultural sensitivity, and cultural competence.

The scale is theoretically based on a pathway model that focuses on the interaction between nursing faculty, and students with different backgrounds. The CAS was developed for use by nursing faculty and nursing students and has been used in several studies for measuring cultural awareness among these groups [13, 16, 17]. The CAS has been tested for and translated into several different languages, including Swedish [18], Korean [19] and Turkish [20]. However, whether the CAS is suitable for use in other groups of students in higher education, within the health and social care fields, is still unknown.

## Methods

This study aimed to test the psychometric properties of a modified version of the CAS (mCAS) for use in higher education within the health and social care fields.

This study was performed in two stages. In stage one, a modified version of the CAS was developed. This version was tested psychometrically in stage two, using cross-sectional data.

### Cultural awareness scale

The CAS was originally developed in English for nursing students and consists of 36 items with responses based on a 7-point scale (ranging from “strongly disagree” to “strongly agree”). There is also one additional alternative response: “does not apply.” Lower scores indicate less cultural awareness, and higher scores indicate greater awareness. Cultural awareness is considered to be the minimal level of cultural competence. Therefore, the CAS evaluates nursing student performance on the first stage of cultural competence development [6]. The scale was initially tested with undergraduate and graduate nursing students in the United States. A content validity index of 0.88 was obtained, and the internal consistency varied from 0.94–0.71. An exploratory factor analysis was performed, and five subscales emerged: “general educational experience,” “cognitive awareness,” “research issues,” “behaviors/comfort with interactions,” and “patient care/clinical care” [6]. These five factors explained 51% of the variance in scale scores. However, during a reanalysis of the CAS, Rew et al. [16] found evidence for a valid three-factor solution (“general attitudes,” “research attitudes,” “clinical experience”), and the analysis supported the reliability of the CAS.

### Stage one

The first stage involved careful review of the wording of each of the 36 statements in the original English version of the CAS by the first and last authors. Wordings that referred to nursing and nursing school were changed into terms that are more applicable to a broader group of students in educational programs within the health and social care fields. For example, “The instructors at this nursing school adequately address multicultural issues in nursing” was changed to “The instructors at this university adequately address multicultural issues.” Further, the word “patient” was replaced with the word “client” throughout the scale. Item number 20 in the original CAS was excluded because this statement focused on nursing instructors in clinical settings, a specific nursing educational context. The entire research team reviewed the modified scale and decided on the final version.

The mCAS consists of 35 items, and, similar to the original version, its responses are based on a 7-point scale, ranging from “strongly disagree” to “strongly agree.” The additional alternative response “does not apply” is also used in the modified version.

### Stage two

Stage two involved empirical testing of the mCAS in a cross-sectional survey performed at Malmö University (in Sweden) and Hong Kong Polytechnic University.

### Study sample

A total of 191 undergraduate students, from the fields of social care (*n* = 18), dental hygiene (*n* = 30), criminology (*n* = 40), occupational therapy and physiotherapy (*n* = 103), responded to the survey. Of all the respondents, 88 were students at Malmö University, and 103 were students at Hong Kong Polytechnic University. The mean age of the students was 23 years (SD 4.27), and 67 (37%) were men.

### Data collection

After permission from the head of each department, contact with the students’ teachers was made, and arrangements for when and how data collection would be performed was decided. In most cases, data were collected in connection to mandatory lectures. All participating students provided written informed consent after receiving oral and written information about the study, including the information that participation in the study was voluntary. The study was approved by the Regional ethical review board, Lund Sweden (No 2017/198) and the institutional research ethics board at Hong Kong Polytechnic University (HSEARS20170227002–0).

### Analysis

Descriptive statistics were used in the analysis of age, gender, type of undergraduate program, and mCAS scores were reported as frequencies, percentages, means, standard deviations, median values, and interquartile ranges. The validity of the mCAS was tested by using an exploratory factor analysis (*n* = 170). The Kaiser-Meyer-Olkin (KMO) Test was used to measure sampling adequacy and the appropriateness of continuing the factor analysis (KMO > 0.60) [21]. A Bartlett’s test of sphericity was performed to analyze the overall significance of correlations within a matrix (*p*-value < 0.05) [21]. A scree plot was performed to graphically determine the optimal number of factors to retain [22]. In the next step, a principal component analysis with varimax rotation was used for the creation of factors. Generally, factor loadings > 0.50 are recommended; however, loadings > 0.30 are considered acceptable [21]. The cut-off criterion of eigenvalue > 1.0 was used for selection of factors. Cronbach’s alpha coefficients were calculated to assess the internal consistency of the total scale and subscales. The Pearson correlation coefficient was analyzed for item-scale correlation. Data analysis was performed using the statistical package SPSS 24.0 (IBM Corporation, Armonk, NY, USA).

## Results

The mCAS appeared to be easy to answer, and few missing items were identified. However, for some of the items, the “does not apply” response, was chosen more frequently. For example, 16 students chose this response for item 16 (“In classes, my instructors/supervisors have engaged in behaviors that may have made students from certain cultural backgrounds feel excluded”). Fourteen students thought that item 13 (“I have noticed that the instructors/supervisors at this university call on students from minority cultural groups when issues related to their group come up in class”) did not apply. This affected the sample size in the final factor analysis.

The factor analysis was performed on data from 170 students who had completed the mCAS and answered all items. The internal dropout rate was 11% (21 students with incomplete questionnaires). The initial exploratory factor analysis showed good sampling adequacy with a KMO value of 0.738 and a statistically significant Bartlett’s sphericity (x^2^ 2384.231 *p*-value < 0.001). Ten components showed an eigenvalue > 1.0, explaining 65.28% of the total variance. When analyzing the scree plot, a clear cut was seen at four components, and four was determined to be the optimal number of factors to retain. A principal component factor analysis with varimax rotation was performed to analyze the four-factor model. It is preferable that each item load on only one factor. However, five items (10, 15, 21, 24, and 25) loaded on multiple factors, and these items were then placed into the most relevant factor group. Then the final factor model of the mCAS was constructed (Table 1). The four factors explained 43.4% of the total variance. All items had a factor loading > 0.3. Factor 1, “general educational and research experience,” included 15 items and was the strongest factor (16.87% of the total variance). Factor 2, “behaviors/comfort with interactions,” included 8 items. Factor 3, “cognitive awareness,” and factor 4, “clinical issues” included 7 and 5 items, respectively (Table 1).

**Table 1:** Factor loadings for the modified Cultural Awareness Scale (*N* = 170)

Item	Factor 1 General educational and research experience	Factor 2 Behaviors/ comfort with interactions	Factor 3 Cognitive awareness	Factor 4 Clinical Issues
1 The instructors at this university adequately address multicultural issues	.717			
2 This university provides opportunities for activities related to multicultural issues.	.626			
3 Since entering this university my understanding of multicultural issues has increased.	.578			
4 My experiences at this university have helped me become knowledgeable about the health problems associated with various racial and cultural groups	.620			
5 I think my *beliefs and attitudes* are influenced by my culture.			.725	
6 I think my *behaviors* are influenced by my culture.			.851	
7 I often reflect on how culture affects beliefs, attitudes, and behaviors.			.553	
8 When I have an opportunity to help someone, I offer assistance less frequently to individuals of certain cultural backgrounds.		.699		
9. I am less patient with individuals of certain cultural backgrounds		.635		
10 I feel comfortable working with patients/clients of all ethnic groups.		.342		
11 I believe healthcare/social work professionals own cultural beliefs influence their care decisions.			.415	
12 I typically feel somewhat uncomfortable when I am in the company of people from cultural or ethnic backgrounds different from my own.		.684		
13 I have noticed that the instructors/supervisors at this university call on students from minority cultural groups when issues related to their group come up in class.		.622		
14 During group discussions or exercises, I have noticed the instructors/supervisors make efforts to ensure that no student is excluded.	.529			
15 I think that students’ cultural values influence their classroom behaviors (for example, asking questions, participating in groups, or offering comments.)			.366	
16 In my classes, my instructors/supervisors have engaged in behaviors that may have made students from certain cultural backgrounds feel excluded.		.739		
17 I think it is the instructor’s/supervisor’s responsibility to accommodate the diverse learning needs of students.			.455	
18 My instructors/supervisors at this university seem comfortable discussing cultural issues in the classroom.	.427			
19 My instructors/supervisors seem interested in learning how their classroom behaviors may discourage students from certain cultural or ethnic groups.	.524			
20 I believe the classroom experiences at this university help our students become more comfortable interacting with people from different cultures.	.572			
21 I believe that some aspects of the classroom environment at this university may alienate students from some cultural backgrounds.		.557		
22 I feel comfortable discussing cultural issues in the classroom				.408
23 My courses at this university have helped me become more comfortable interacting with people from different cultures.	.581			
24 I feel that this university’ instructors/supervisors respect differences in individuals from diverse cultural backgrounds.	.419			
25 The instructors/supervisors at this university model behaviors that are sensitive to multicultural issues.			.325	
26 The instructors/supervisors at this university use examples and/or case studies that incorporate information from various cultural and ethnic groups	.614			
27 The faculty at this university conducts research that considers multicultural aspects of health-related issues.	.714			
28 The students at this university have completed theses and dissertation studies that considered cultural differences related to health issues.	.617			
29 The researchers at this university consider relevance of data collection measures for the cultural groups they are studying.	.717			
30 The researchers at this university consider cultural issues when interpreting findings in their studies.	.684			
31. I respect the decisions of my patients/clients when they are influenced by their culture, even if I disagree.				.575
32 If I need more information about a patient’s/client’s culture, I would use resources available on site (for example, books, videos, etc.).				.638
33 If I need more information about a patient’s/client’s culture, I would feel comfortable asking people I work with				.830
34 If I need more information about a patient’s/ client’s culture, I would feel comfortable asking the patient/client or a family member.				.746
35 I feel somewhat uncomfortable working with the families of patients/clients from cultural backgrounds different than my own.		.641		
Eigen value	5.904	3.756	2.786	2.743
Percentage of variance	16.87	10.73	7.96	7.84

### Reliability analysis

The mean total scale score was 163.5 with a standard deviation of 25.5. The mean item score for all items was 4.66, ranging from 3.78 to 5.61. Internal consistency was analyzed for the total mCAS and for each of the four subscales identified in the factor analysis. Sample size varied across the subscales due to individual missing data. The Cronbach’s alpha value was 0.88 for the total scale and ranged from 0.7 (“cognitive awareness”) to 0.9 (“general educational and research experience”) for the four subscales (Table 2).

**Table 2:** Internal consistency and average item for the modified Culture Awareness Scale and subscales

Subscale	Item Mean	Scale Mean (SD)	n	No of items	Cronbach’s alpha
General educational and research experience (GERE)	4.369 (3.777–5.151)	65.53 (14.29)	179	15	.90
Behaviors/comfort with interactions (BCI)	4.849 (4.176–5.543)	38.79 (10.26)	187	8	.83
Cognitive awareness (CA)	4.777 (4.189–5.395)	33.44 (5.96)	190	7	.70
Clinical Issues (CI)	5.051 (4.899–5.333)	25.25 (6.36)	189	5	.82
Total scale	4.664 (3.778–5.608)	163.25 (25.5)	176	35	.88

The intercorrelations between the four subscales showed significant correlations between “general educational and research experience,” “cognitive awareness,” and “clinical issues” and between “behaviors/comfort with interactions” and “cognitive awareness” (Table 3).

**Table 3:** Correlations between the four subscales of the modified Culture Awareness Scale

Subscale	General educational and research experience	Behaviors/comfort with interactions	Clinical Issues	Cognitive awareness
General educational and research experience	1.0	.079	.427^a^	.351^a^
Behaviors/comfort with interactions		1.0	−.059	.245^a^
Clinical Issues			1.0	.225^a^
Cognitive awareness				1.0

^a^Correlation is significant at the 0.01 level

## Discussion

The results show that the mCAS is a four-factor measure of cultural awareness with satisfactory reliability in terms of internal consistency. This finding suggests that the mCAS may be used as a generic tool to measure cultural awareness among students in higher education within the health and social care fields.

The factor analysis supported a four-factor structure of the mCAS. Similar findings were seen in a study that tested a Turkish version of the CAS [20], which also resulted in a four-factor solution. However, the items loaded slightly differently from the present study. For example, all items loaded in factor four of the Turkish study were different than those in factor four of the present study. The four-factor solution contrasts with the original five-factor structure [6] that was further confirmed in the Swedish version [18]. A five-factor structure solution was also confirmed by Oh et al. [19] who tested a Korean version of the CAS. However, the subscales were divided differently from the original CAS, and the subscale “general educational experience” was divided between factors two and four. Moreover, a confirmatory factor analysis in the reanalysis of the CAS provided evidence for a three-factor measure of cultural awareness [16]. This illustrates how construct validity is an ongoing process and emphasizes the importance of investigating psychometric properties for established measurements before further use in other cultural settings or target populations. In particular, establishing construct validity is important in the assessment of complex phenomenon such as cultural awareness.

In the present study, five items loaded on multiple factors. It is recognized that this may have aggravated interpretation of the factors [23, 24]. A similar pattern was seen in the testing of the Turkish version of the CAS [20] and in a replication of the original study [6], conducted by Krainovich et al. [13]. However, whether it concerned the same items as in our study is unknown. These findings contradicted those of studies on the original scale [6], and the Swedish [18] and Korean versions [19], neither of which reported any cross-loading items. Nevertheless, it is quite common for items to load significantly on multiple factors, and, as recommended by Pett et al. [21], these items were placed into the most relevant factor group.

The internal consistency was moderate to excellent for all the subscales and the total mCAS, suggesting strong intercorrelations between the scale items. This was also the case for the original CAS [6] and in re-analyses of the CAS [13, 16]. However, both the Swedish [18] and Korean [19] versions showed poor alpha values for the factor “behavior/comfort with interactions.” For the mCAS this factor included eight items, which is two more than in the original version, and this may be one reason for the high internal consistency. These two items were on instructor behavior that may have made students from certain cultural background feel excluded (item 16) and aspects in the classroom environment that may alienate students from some cultural backgrounds (item 21). This suggests that the factor “behavior/comfort with interactions” included not only the ability to interact with others from different cultures but also external aspects that may influence interactions. It is worth mentioning that seven of the eight items included in the subscale “behavior/comfort with interactions” are worded negatively and were re-coded in the analysis, which in turn may have attenuated internal consistency [25].

Analysis of the intercorrelations between the four subscales showed some significant correlations. However, correlation coefficients were low (*r* = < 0.5) [26], and a negative correlation was seen between “behavior/comfort with interactions” and “clinical issues.” This corresponds to previous studies that tested the CAS [6, 18, 19], except for higher correlations between the subscales “cognitive awareness” and “clinical issues” and between “general educational experience” and “research issues” in the Korean version [19]. However, it is difficult to compare the different studies because the items loaded somewhat differently in the factor analyses. We can assume that some dimensions of cultural awareness are related and can therefore expect some of the subscales to be correlated. Nevertheless, a very high correlation between two scales may imply that the scales are measuring the same factor, and there is a need to consider whether they could be combined into one single scale [27]. The low intercorrelations between the subscales of the mCAS may therefore be acceptable and may suggest a satisfactory convergent validity.

One possible limitation of the study may be the sample size. However, determining the sample size for factor analysis is challenging. Varying opinions and guidelines exist in the literature [28]. Previous studies have suggested that an adequate sample size for a factor analysis is partly determined by the nature of the data [24, 28]. In fact, it has been demonstrated that sample sizes can be rather small when the communalities are high (>.60) and each factor is defined by several items [24, 28, 29]. In the present study, all communalities were high, and at least five items defined the factors, suggesting that the sample size was satisfactory.

In both the original CAS and mCAS, responses to the question items are based on a 7-point scale with a midpoint choice, which is “no opinion,” and one additional alternative response, which is “does not apply.” The alternative response “does not apply” was chosen by several of the students, which was one reason for incomplete questionnaires. Some researchers argue that “don’t know or no opinion” alternatives lead to incomplete, less valid, and less informative data, whereas others maintain that it means that there is no evidence to support such impact [30]. In the case of the mCAS, it may be preferable to only have the midpoint choice “no opinion”; however, the effects of deleting the alternative response “does not apply” must be further tested and evaluated.

The mCAS exists only in English; however, most of the students were not native English speakers, which could contribute to misunderstandings and misinterpretations when responding to the questionnaire. We do not fully understand their level of English proficiency, including their ability to read, write, speak, and listen in English. However, one eligibility requirement for studies in higher education both in Sweden and Hong Kong is sufficient grades in English language courses taken in upper secondary school.

## Conclusion

The mCAS showed satisfactory psychometric properties and can be recommended as a generic tool to measure cultural awareness among students in higher education. This might be particularly important as interprofessional collaboration is gaining momentum in health care education. The modified scale may thus be useful when evaluating effectiveness of curriculum and educational interventions aimed to improve cultural safety among students within the health and social care fields. However, to be able to use the mCAS to ensure the efficacy of cultural awareness interventions, further psychometric testing is needed. Studies with a higher number of students are warranted, the ability to detect differences between groups and the responsiveness of the scale should be tested to establish the mCAS’s ability to reflect changes in cultural awareness over time.

## Data Availability

The data generated and analysed during the current study are available from the corresponding author upon reasonable request.

## Abbreviations

CAS: Cultural Awareness Scale
KMO: The Kaiser-Meyer-Olkin
mCAS: Modified version of the Cultural Awareness Scale
